# Ninjurin2 overexpression promotes glioma cell growth

**DOI:** 10.18632/aging.102515

**Published:** 2019-12-02

**Authors:** Li-Na Zhou, Ping Li, Shang Cai, Gang Li, Fang Liu

**Affiliations:** 1 Department of Radiotherapy and Oncology, Affiliated Kunshan Hospital of Jiangsu University, Kunshan, China; 2 Department of Radiotherapy and Oncology, The Second Affiliated Hospital of Soochow University, Suzhou, China; 3 Department of Chemoradiation Oncology, The First affiliated Hospital of Wenzhou Medical University, Wenzhou, China; 4 Department of Neurosurgery, Nanjing Medical University Affiliated Changzhou No. 2 People’s Hospital, Changzhou, Jiangsu, China

**Keywords:** glioma, Ninj2, Akt, receptor tyrosine kinases

## Abstract

Ninjurin2 (Ninj2) is an adhesion protein expressed in neurons and glial cells. The current study tested its expression and potential functions in human glioma. We show that *Ninj2 mRNA* and protein levels are significantly upregulated in human glioma cells and tissues. In established and primary human glioma cells, Ninj2 shRNA or knockout (by CRISPR/Cas9 gene editing) potently inhibited cell survival, growth, proliferation, cell migration and invasion, while inducing apoptosis activation. Contrarily, ectopic overexpression of Ninj2 promoted glioma cell progression *in vitro*. In human glioma tissues and cells, Ninj2 co-immunoprecipitated with multiple receptor tyrosine kinases (EGFR, PDGFRβ and FGFR), required for downstream Akt and Erk activation. Akt and Erk activation was potently inhibited by Ninj2 shRNA or knockout, but enhanced with ectopic Ninj2 overexpression in glioma cells. In summary, we show that Ninj2 overexpression promotes glioma cell growth.

## INTRODUCTION

Glioma is a common brain malignancy, causing significant mortalities each year [1–3]. High-grade (grade III-IV) gliomas (*i.e.* glioblastoma) have one worse prognosis among all human malignancies [1–3]. Current treatment options for this devastating disease, including surgical tumor resection, radiation therapy and/or temozolomide-based chemotherapy, have failed to significantly improve the overall survival for advanced gliomas [4–6].

The molecular heterogeneity are commonly detected in human gliomas [1], leading to dysregulation and overactivation of multiple signaling cascades [7–9]. These cascades, including PI3K-Akt-mTOR and Erk-MAPK signaling, shall promote glioma tumorigenesis and progression [7–9]. The molecularly targeted therapy has therefore become the research focus for glioma therapies [7–11].

Ninjurin2 (Ninj2), a homolog of ninjurin1 (Ninj1) [12], is a novel adhesion protein in neurons and glial cells [12]. Ninj2 and Ninj1 have the same conserved hydrophobic regions in the transmembrane domains. Their adhesion motifs are, however, different [12]. *Ninj2* gene is located on chromosome 12p13 [12]. An early genome-wide association study has reported that two *Ninj2* single nucleotide polymorphisms (SNPs, rs11833579 and rs12425791) are associated with ischemic stroke in Caucasians [13]. Although inconsistent results have been reported by the following studies [14–18].

Jing et al. have shown that Ninj2 could inhibit oxidative stress-induced injury to neuronal cells [19]. Additionally, Liu et al. demonstrated four-octyl itaconate (4-OI) increased Ninj2 expression and protected neuronal cells from hydrogen peroxide [20]. These results highlighted a key pro-survival activity of Ninj2 in neuronal cells [19, 20]. Studies have also found that Ninj2 participates in endothelial inflammation and activation, regulating atherosclerosis progression [21]. The expression and potential functions of Ninj2 in human glioma have not been extensively studied. Here our results will show that overexpression of Ninj2 promotes human glioma cell progression.

## RESULTS

### Ninj2 is upregulated in human glioma cells and tissues

**Figure 1:**
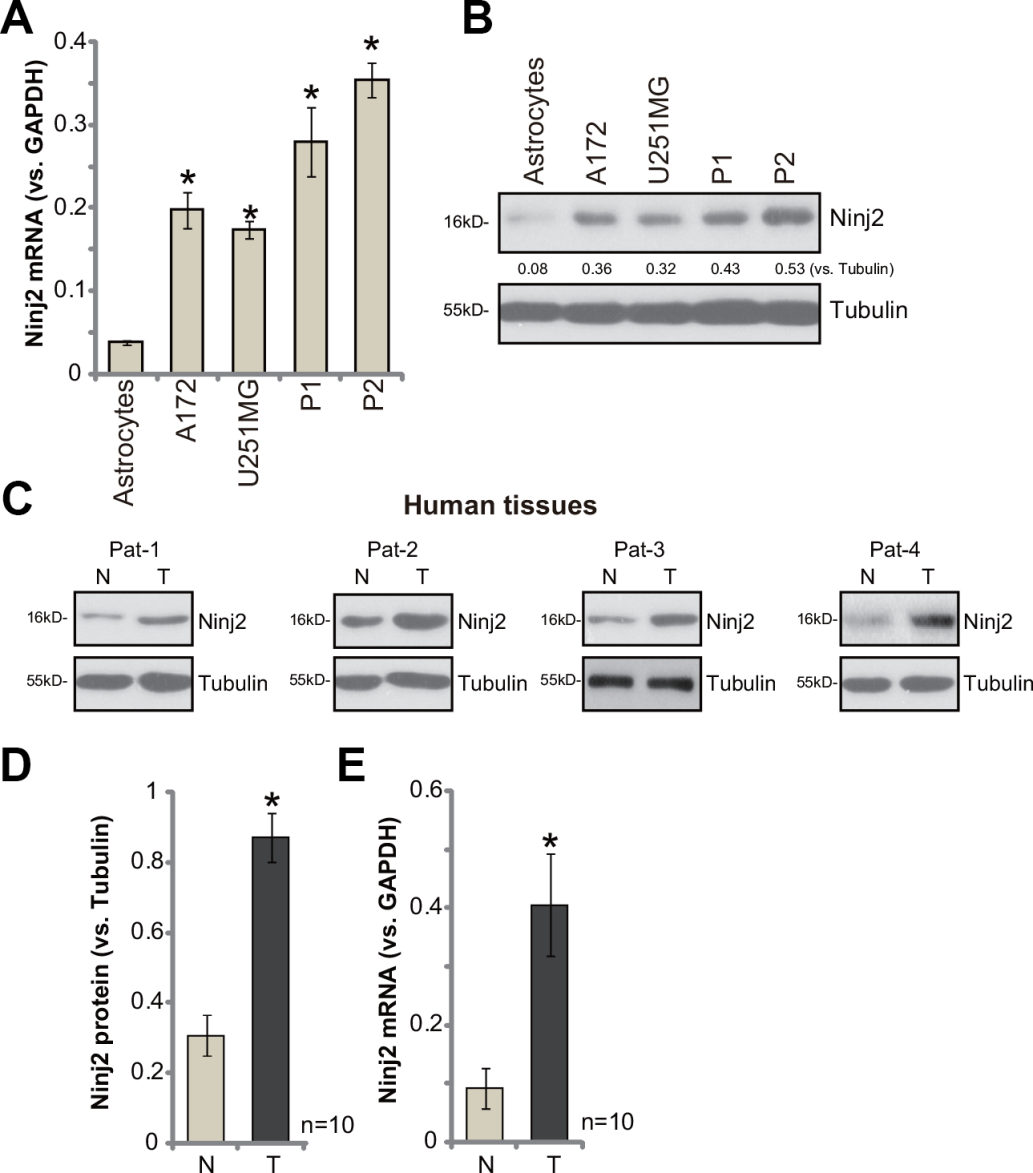
**Ninj2 is upregulated in human glioma cells and tissues.** *Ninj2 mRNA* and protein levels in established (A172 and U251MG) and primary human (“P1/P2”) glioma cells as well as in the primary human astrocytes (“Astrocytes”) were tested by qPCR (**A**) and Western blotting (**B**), respectively. A total of ten (10) pairs of human glioma tissues (“T”) and paired surrounding normal brain tissues (“N”) were homogenized and dissolved in tissue lysis buffer, *Ninj2 mRNA* and protein expression was tested (**C**–**E**). Data were presented as the mean ± SD (same for all Figures).*p<0.05 vs. “Astrocytes” (**A**). *p<0.05 vs. “N” tissues (**D** and **E**). Experiments in this figure were repeated four times, and similar results were obtained.

First, we tested expression of Ninj2 in human glioma cells. As compared to the primary human astrocytes (from Dr. Cao at Soochow University [11]), *Ninj2 mRNA* levels were significantly elevated in established human glioma cell lines (A172 and U251MG) and primary human glioma cells (derived from two human patients, “P1/P2” [11]) (Figure 1A). Ninj2 protein levels were upregulated as well in glioma cells (Figure 1B). Ninj2 protein upregulation was detected as well in human glioma tissues (“T”, Figure 1C and 1D), whereas its levels are relatively low in the paired surrounding normal brain tissues (“N”, Figure 1C and 1D). In the glioma tissues *Ninj2 mRNA* upregulation was also detected (Figure 1E). These results confirm that Ninj2 is upregulated in human glioma cells and tissues, indicating a potential function of Ninj2 in promoting glioma cell progression.

### Ninj2 shRNA or KO inhibits human glioma cell survival

**Figure 2:**
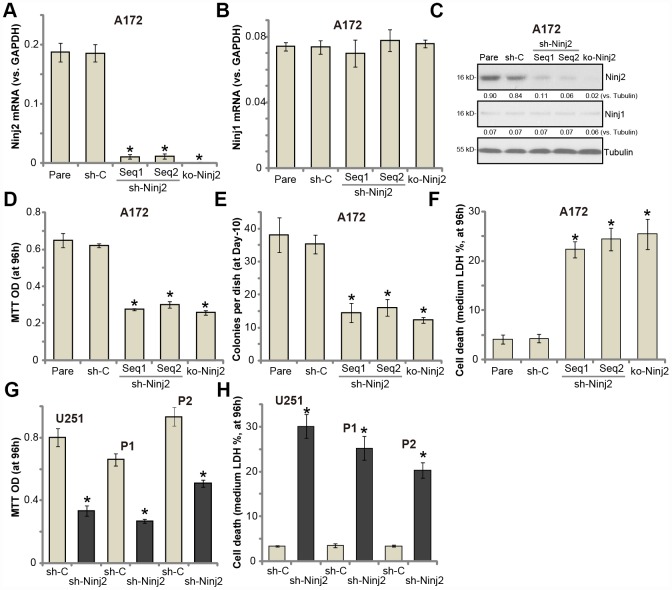
**Ninj2 shRNA or KO inhibits human glioma cell survival.** A172 glioma cells (**A**–**F**), U251MG glioma cells (**G** and **H**) or the primary human glioma cells (derived two patients, “P1/P2”, G and H) were transduced with lentiviral Ninj2 shRNAs (“sh-Ninj2”, two different sequences “Seq1/Seq2”), control shRNA (“sh-C”) or the CRISPR/Cas9 Ninj2 KO construct (“ko-Ninj2”), stable cells were established via puromycin selection; Expression of listed genes was tested by qPCR and Western blotting (**A**–**C**); Cell survival (**D** and **G**), soft agar colony formation (**E**) and cell death (**F** and **H**) were tested by appropriate assays. Ninj2 and Ninj1 proteins were quantified and normalized to the loading control (**C**). For each assay, n=5. For all the functional assays exact same number of viable cells of different genetic manipulations were initially seeded into each well/dish (At 0h or Day0) (same for all Figures). “Pare” stands for the parental control cells (same for all Figures). *p<0.05 vs. “Pare” cells. Experiments in this figure were repeated four times, and similar results were obtained.

To study the potential function of Ninj2 in glioma cells, two lentiviral Ninj2 shRNAs, with non-overlapping sequences (namely “Seq1/Seq2”), were individually transduced to A172 cells. With selection by the puromycin, the stable cell lines were established (“sh-Ninj2” cells). Furthermore, the lentivirus with the lenti-CRISPR/Cas9 Ninj2 KO construct was transduced to A172 cells, establishing the Ninj2 KO stable A172 cells (“ko-Ninj2” cells). In the stable cells with Ninj2 shRNA and Ninj2 KO construct *Ninj2 mRNA* levels decreased significantly (over 95% vs. control cells) (Figure 2A). *Ninj1 mRNA* levels were however unchanged (Figure 2B). Ninj2 protein levels were also significantly downregulated in “sh-Ninj2” cells and “ko-Ninj2” A172 cells (Figure 2C), where the Ninj1 protein expression unchanged (Figure 2C).

Recent studies have indicated an important function of Ninj2 in neuronal cell survival [19, 20]. Testing cell viability, by MTT assay, demonstrated that Ninj2 shRNA or KO resulted in significant viability reduction in A172 cells (Figure 2D). Furthermore, Ninj2 silencing/KO decreased the number of viable A172 cell colonies (Figure 2E). Furthermore, a significant increase of medium LDH release was detected in “sh-Ninj2” and “ko-Ninj2” A172 cells (Figure 2F), indicating cell death. In U251MG cells and the primary human glioma cells (“P1/P2”), transfection of Ninj2 shRNA (“Seq1”) lentivirus resulted in significant viability (MTT OD) reduction (Figure 2G) and cell death (LDH release, Figure 2H). The control shRNA virus (“sh-C”) failed to inhibit Ninj1/2 expression and glioma cell functions (Figure 2A–2H). Together, these results show that Ninj2 shRNA or KO inhibited glioma cell survival.

### Ninj2 shRNA or KO inhibits human glioma cell proliferation

**Figure 3:**
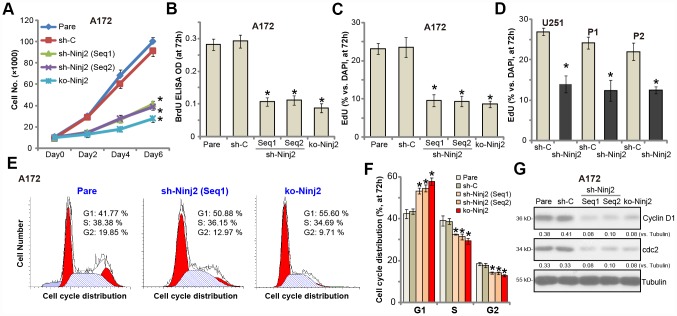
**Ninj2 shRNA or KO inhibits human glioma cell proliferation.** A172 glioma cells (**A**–**C**, **E**–**G**), U251MG glioma cells (**D**) or the primary human glioma cells (derived two patients, “P1/P2”, D) were transduced with lentiviral Ninj2 shRNAs (“sh-Ninj2”, two different sequences “Seq1/Seq2”), control shRNA (“sh-C”) or the CRISPR/Cas9 Ninj2 KO construct (“ko-Ninj2”), stable cells were established via puromycin selection; Cell proliferation was examined by the assays mentioned in the text (**A**–**D**), and cell cycle progression tested by PI-FACS (**E** and **F**), with cyclin D1 and cdc2 expression tested and quantified (**G**). For each assay, n=5. *p<0.05 vs. “Pare” cells. Experiments in this figure were repeated four times, and similar results were obtained.

To test the potential effect of Ninj2 on glioma cell proliferation, we utilized the cell counting assay. As shown, the stable A172 cells with Ninj2 shRNA (“Seq1/Seq2”) or the KO construct (see Figure 2) grew significantly slower than control cells (Figure 3A). Furthermore, we show that Ninj2 shRNA/KO potently inhibited BrdU incorporation in A172 cells (Figure 3B). Additionally, EdU-positive staining in A172 glioma cells was largely decreased after Ninj2 silencing or KO (Figure 3C). These results clearly show that Ninj2 shRNA or KO inhibited A172 cell proliferation. In U251MG cells and primary human glioma cells (“P1/P2”), Ninj2 shRNA similarly decreased EdU incorporation, suggesting proliferation inhibition (Figure 3D). PI-FACS assay results demonstrated that Ninj2 silencing or KO disrupted A172 cell cycle progression, causing G1-S arrest (Figure 3E and 3F). Expression of cell cycle-associated proteins, including cyclin D1 and cdc2, was also downregulated in Ninj2-silenced/-KO A172 cells (Figure 3G). The control shRNA virus (“sh-C”) had no significant effect on proliferation and cell cycle progression of glioma cells (Figure 3A–3G).

### Ninj2 shRNA or KO inhibits human glioma cell migration and invasion

**Figure 4:**
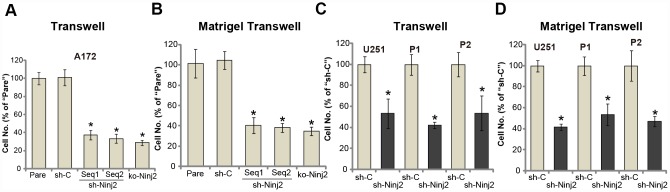
**Ninj2 shRNA or KO inhibits human glioma cell migration and invasion.** A172 glioma cells (**A** and **B**), U251MG glioma cells (**C** and **D**) or the primary human glioma cells (derived two patients, “P1/P2”, C and D) were transduced with lentiviral Ninj2 shRNAs (“sh-Ninj2”, two different sequences “Seq1/Seq2”), control shRNA (“sh-C”) or the CRISPR/Cas9 Ninj2 KO construct (“ko-Ninj2”), stable cells were established via puromycin selection; Cells were subjected to Transwell assays (**A** and **C**) and Matrigel Transwell assays (**B** and **D**) (both for 24h), results were quantified. For each condition, at least 1000 cells in five random views (magnification : 1×200) were included to calculate the average number of migrated/invasive cells. For each assay, n=5. *p<0.05 vs. “Pare” cells. Experiments in this figure were repeated four times, and similar results were obtained.

Uncontrolled glioma cell migration and invasion are essential for glioma cell progression [1, 22]. Ninj2 is an adhesion protein in neurons. We therefore tested whether Ninj2 was important for glioma cell migration and invasion. Performing the “Transwell” assays, we show that A172 cell *in vitro* migration was significantly inhibited by Ninj2 shRNA (“Seq1/Seq2”) and KO (Figure 4A). Furthermore, testing cell invasion, by the “Matrigel Transwell” assays, demonstrated that Ninj2 silencing or depletion dramatically attenuated A172 cell invasion as well (Figure 4B). The control shRNA virus (“sh-C”), as expected, exerted no significant effect on A172 cell migration and invasion (Figure 4A and 4B). In U251MG cells and the primary human glioma cells (“P1/P2”), “Transwell” and “Matrigel Transwell” assay results show that Ninj2 shRNA significantly decreased the number of migrated (Figure 4C) and invasive (Figure 4D) glioma cells. Taken together, Ninj2 shRNA or KO inhibited human glioma cell migration and invasion *in vitro*.

### Ninj2 shRNA or KO induces apoptosis activation in human glioma cells

**Figure 5:**
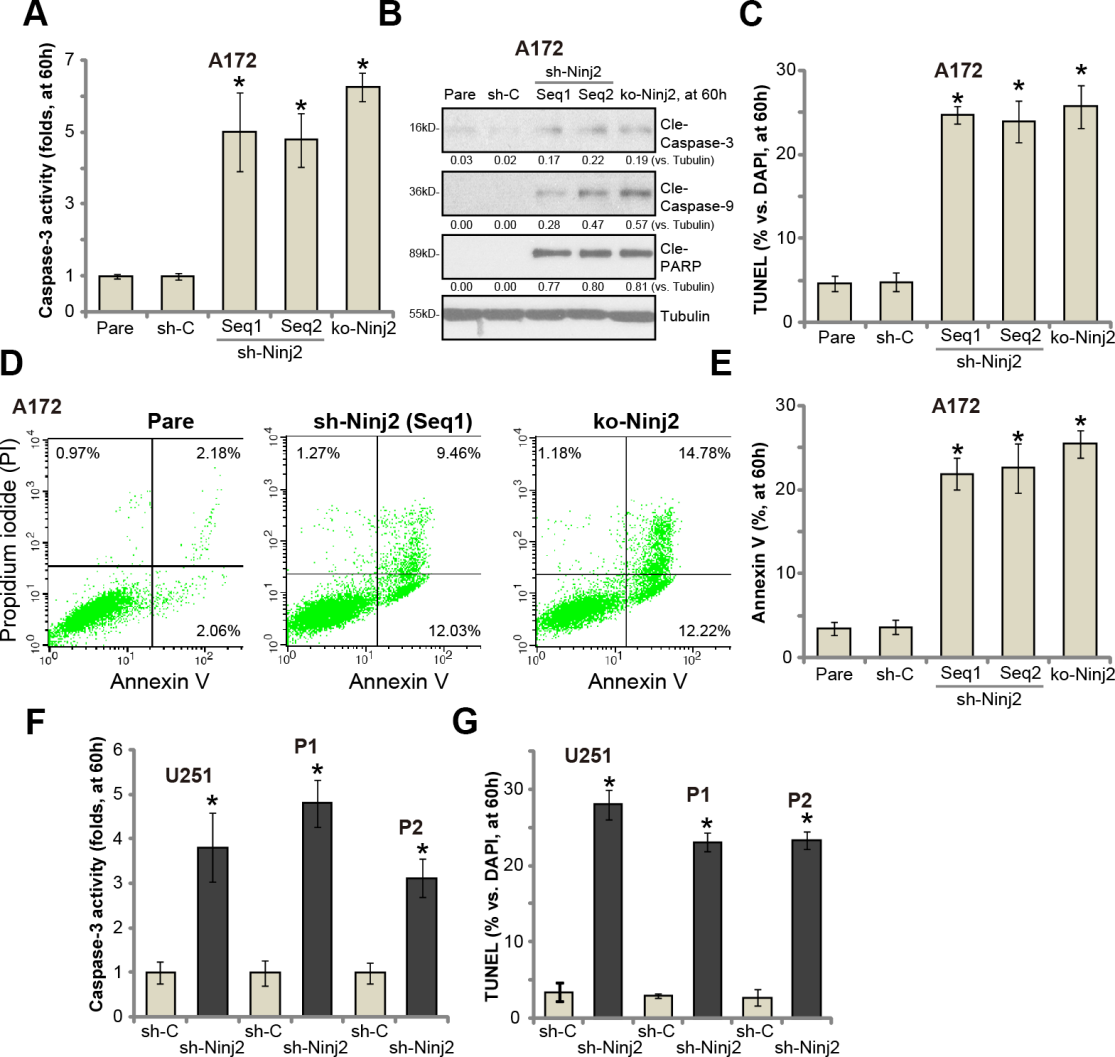
**Ninj2 shRNA or KO induces apoptosis activation in human glioma cells.** A172 glioma cells (**A**–**E**), U251MG glioma cells (**F** and **G**) or the primary human glioma cells (derived two patients, “P1/P2”, **F** and **G**) were transduced with lentiviral Ninj2 shRNAs (“sh-Ninj2”, two different sequences “Seq1/Seq2”), control shRNA (“sh-C”) or the CRISPR/Cas9 Ninj2 KO construct (“ko-Ninj2”), stable cells were established via puromycin selection; The relative caspase-3 activities were tested (**A** and **F**); Cell apoptosis was tested by TUNEL staining (**C** and **G**) and Annexin V FACS (**D** and **E**); Expression of listed proteins was tested by Western blotting (**B**). Expression of listed proteins was quantified and normalized to the loading control (**B**). For each assay, n=5. *p<0.05 vs. “sh-C” cells. Experiments in this figure were repeated five times, and similar results were obtained.

Apoptosis activation could be one reason of glioma cell death and proliferation inhibition. As shown in Figure 5A, the caspase-3 activity was significantly increased in Ninj2 shRNA- or Ninj2 KO-A172 cells. Western blotting assay results confirmed that Ninj2 shRNA or KO induced cleavages of caspase-3, caspase-9 and ploy ADP ribose polymerase (PARP) in A172 cells (Figure 5B). The TUNEL-positive nuclei ratio was significantly augmented as well (Figure 5C). Furthermore, Ninj2 silencing or KO significantly increased Annexin V-positive staining in A172 cells (Figure 5D and 5E). These results indicated that Ninj2 shRNA/KO induced apoptosis activation in A172 cells. In U251MG cells and the primary human glioma cells (“P1/P2”), Ninj2 shRNA induced significant apoptosis activation, evidenced by increases of caspase-3 activity (Figure 5F) and TUNEL-positive nuclei ratio (Figure 5G).

### Ninj2 associates with multiple receptor tyrosine kinases, required for downstream Akt and Erk activation

**Figure 6:**
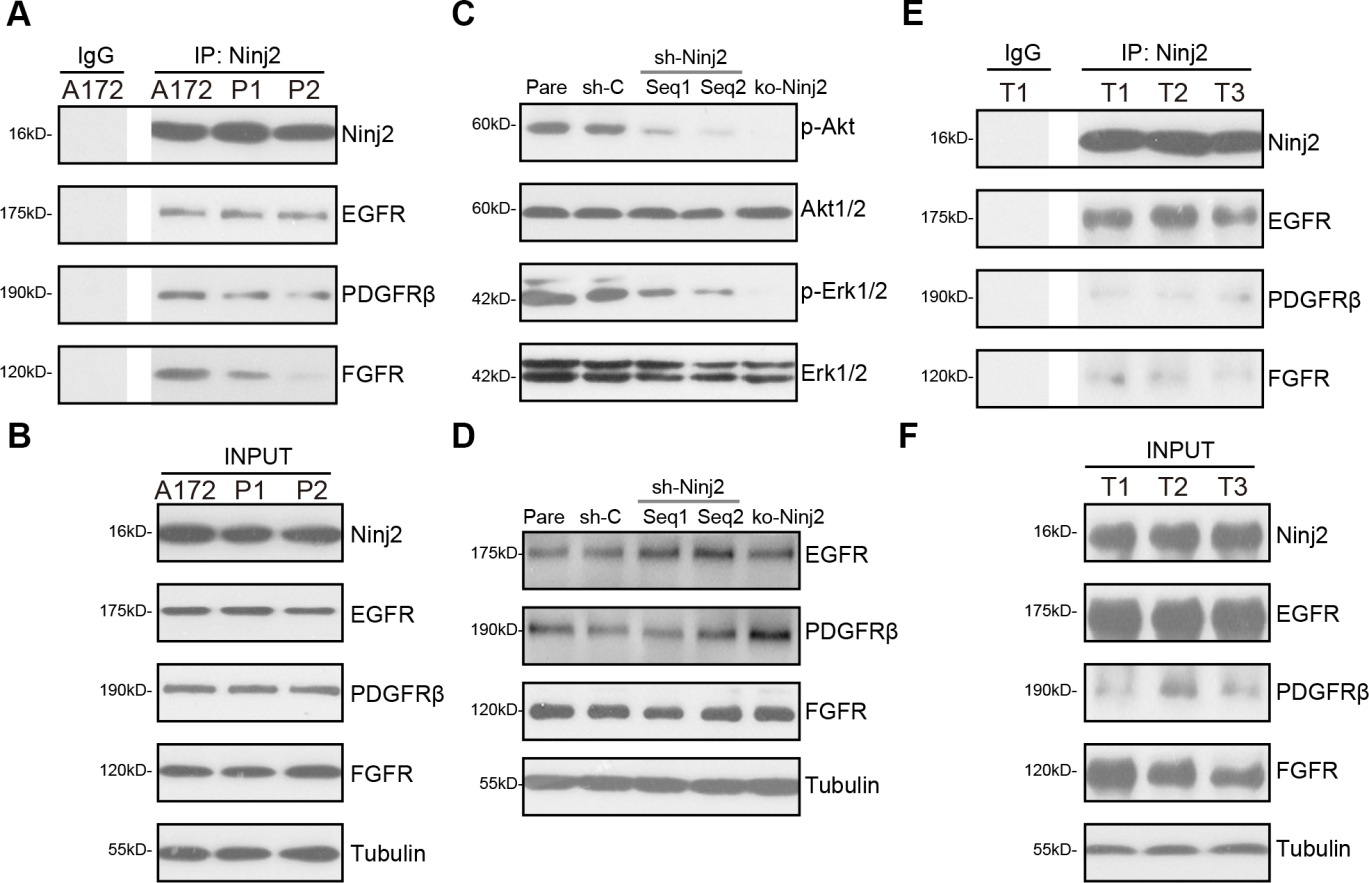
**Ninj2 associates with multiple receptor tyrosine kinases, required for downstream Akt and Erk activation.** In A172 cells and primary human glioma cells (“P1/P2”) the association between Ninj2 with RTKs (EGFR, PDGFRβ and FGFR) was tested by co-immunoprecipitation (“Co-IP”) assays (**A**); “INPUT” shows expression of the RTKs and Ninj2 in total cell lysates (**B**). A172 cells were transduced with lentiviral Ninj2 shRNAs (“sh-Ninj2”, two different sequences “Seq1/Seq2”), control shRNA (“sh-C”) or the CRISPR/Cas9 Ninj2 KO construct (“ko-Ninj2”), stable cells were established via puromycin selection; expression of listed proteins was tested by Western blotting (**C** and **D**); Fresh human glioma tissue lysates from three patients (“T1/2/3”) were subjected to the same Co-IP assay (**E**), “INPUT” shows expression of RTKs and Ninj2 in the tumor lysates (**F**). Experiments in this figure were repeated five times, and similar results were obtained.

Simultaneous activation of several key receptor tyrosine kinase (RTKs) induces sustained and profound activation of downstream cascades, including PI3K-Akt-mTOR and Erk-MAPK, promoting glioma cell progression [11, 23, 24]. RTKs, including EGFR (epidermal growth factor receptor), PDGFRβ (platelet-derived growth factor receptor β), and FGFR (fibroblast growth factor receptor), are key therapeutic targets of glioma [11, 23, 24]. By employing a co-immunoprecipitation (“Co-IP”) assay, we show that Ninj2 immunoprecipitated with EGFR, PDGFRβ and FGFR in A172 cells and primary human glioma cells (“P1/P2”) (Figure 6A). “INPUT” results confirmed Ninj2, EGFR, PDGFRβ and FGFR expression in the glioma cells (Figure 6B).

Significantly, Ninj2 shRNA or KO potently inhibited Akt and Erk activation in A172 cells (Figure 6C), while total Akt1/2 and Erk1/2 (Figure 6C) as well as EGFR, PDGFRβ and FGFR expression (Figure 6D) were unchanged. In the human glioma tissues (“T1/T2/T3”, derived three primary glioma patients), Ninj2-immunoprecipitation with EGFR, PDGFRβ and FGFR was detected as well (Figure 6E). “INPUT” results confirmed Ninj2, EGFR, PDGFRβ and FGFR expression in the glioma tissues (Figure 6F). These results imply that Ninj2 associated with multiple RTKs, required for Akt and Erk activation in glioma cells and tissues.

### Ectopic Ninj2 overexpression promotes glioma cell progression *in vitro*

**Figure 7:**
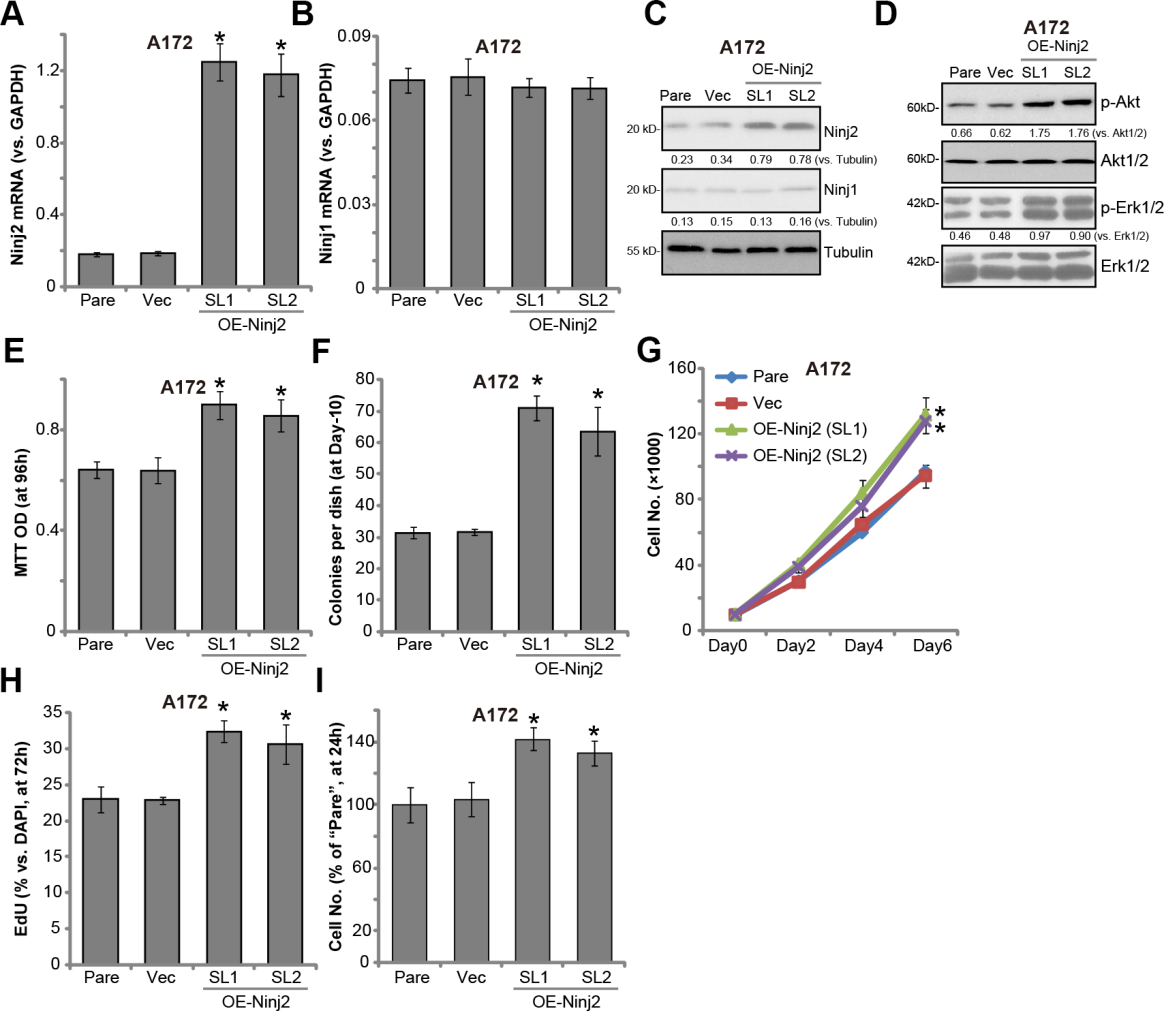
**Ectopic Ninj2 overexpression promotes glioma cell progression *in vitro*.** A172 cells were infected with the Ninj2-cDNA lentivirus (LV-Ninj2), followed by puromycin selection two stable cell lines (“SL1/SL2”) were established (“OE-Ninj2” cells). Control cells were infected with empty vector lentivirus (“Vec”); Expression of listed genes were tested by qPCR and Western blotting (**A**–**D**); Cell viability (MTT OD, **E**) and soft agar colony formation (**F**) were tested; Cell proliferation was tested by cell counting assay (**G**) and EdU staining assay (**H**), with cell migration tested by the Transwell assay (**I**). Expression of listed proteins was quantified and normalized to the loading control (**C** and **D**). For each assay, n=5. *p<0.05 vs. “Vec” cells. Experiments in this figure were repeated three times, and similar results were obtained.

Next, Ninj2-cDNA-expressing lentivirus (LV-Ninj2, from Dr. Zhang [19]) was transduced to A172 cells. Following selection by puromycin, two stable cell lines (“SL1/2”) were established, “OE-Ninj2” cells. qPCR results confirmed that *Ninj2 mRNA* levels increased over 5-6 folds (*vs.* control cells) in “OE-Ninj2” cells (Figure 7A), with *Ninj1 mRNA* unchanged (Figure 7B). Ninj2 protein overexpression was detected as well (Figure 7C). As compared to control cells with empty vector (“Vec”), in “OE-Ninj2” cells Akt and Erk activation was significantly enhanced (Figure 7D). Furthermore, ectopic overexpression of Ninj2 promoted A172 cell survival (Figure 7E), soft agar colony formation (Figure 7F) as well as cell growth (Figure 7G) and EdU staining (Figure 7H). Additionally, A172 cell migration, tested by “Transwell” assays, was enhanced as well in Ninj2-overexpressed cells (Figure 7H). The empty vector did not change Ninj1-Ninj2 expression (Figure 7A–7C), Akt and Erk activation (Figure 7D) and A172 cell functions (Figure 7E–7I). Collectively, these results show that Ninj2 overexpression promoted glioma cell progression *in vitro*.

## DISCUSSION

It has been shown that Ninj2 is an adhesion protein expressed in neurons and glial cells [12, 21]. The potential functions of Ninj2 are largely unknown, although its expression is elevated following nerve injuries [12, 21], promoting neurite outgrowth [12]. A very recent study has suggested that Ninj2 could be an important pro-survival factor in neuronal cells. Ninj2 knockout by CRISPR/Cas9 gene-editing led to SH-SY5Y neuronal cell death and apoptosis. Jing et al. further show that Ninj2 silencing, by its target mRNA miR-764, inhibited neuronal cell survival [19]. Contrarily, ectopic overexpression of Ninj2 can protect SH-SY5Y cells from hydrogen peroxide (H_2_O_2_) [19]. Additionally, Liu et al. have shown that four-octyl itaconate (4-OI), a novel Nrf2 activator, induced Ninj2 expression in human neuronal cells and protected cells against H_2_O_2_ [20].

The results of the present study indicate that Ninj2 could be a novel oncogenic protein for human glioma. Ninj2 is upregulated in human glioma cells and tissues. In established and primary human glioma cells, Ninj2 silencing (by targeted shRNAs) or knockout (by CRISPR/Cas9 method) potently inhibited cell survival, proliferation, migration and invasion, while inducing significant apoptosis activation. Contrarily, forced overexpression of Ninj2 by a lentiviral construct efficiently promoted glioma cell progression *in vitro*.

In human glioma, simultaneous activation of multiple RTKs, including EGFR, VEGFR, FGFR and PDGFR, can constitutively activate key oncogenic downstream cascades, including PI3K-Akt-mTOR and Erk-MAPK signaling [23, 25]. Therefore RTKs are important oncotarget proteins for human glioma [25]. Inhibition or silencing of one RTK could only result in partial or even no inhibition of these downstream cascades and often little anti-glioma actions [23, 25]. Wang et al. have shown that Ninj2 is important for endothelial inflammation and activation, by directly interacting with Toll-like receptor 4 (TLR4) to transduce downstream NF-κB and c-Jun signalings [21]. The results of present study demonstrated that in glioma cells Ninj2 co-immunoprecipitated with multiple RTKs (including EGFR, PDGFRβ and FGFR), required for downstream Akt and Erk activation. In the glioma cells Akt and Erk activation was largely inhibited by Ninj2 shRNA/KO, but augmented with ectopic Ninj2 overexpression.

Our results suggest that fully activation of the oncogenic RTKs (EGFR, PDGFR and FGFR) signaling requires Ninj2 in glioma cells. Ninj2, thought binding directly to the RTKs, should be essential for the downstream signalings (Akt and Erk) transduction. Targeting the Ninj2-RTK signaling will be a potential valuable novel strategy to inhibit glioma cells. The underlying mechanism of Ninj2-mediated RTK signaling transduction should warrant further investigations.

The identification of novel oncogenic proteins is extremely important for the diagnostic and prognostic determination for human glioma. In the current study, we show that expression of Ninj2 is significantly increased in human glioma tissues, as compared to its levels in the surrounding normal brain tissues. These results further support Ninj2 as a valuable therapeutic target of human glioma. Future studies are certainly needed to further explore the significance of Ninj2 upregulation in the diagnosis and therapeutic of human glioma. Collectively, we show that Ninj2 overexpression promotes glioma cell progression, indicating that it could be a novel and valuable therapeutic target for human glioma.

## MATERIALS AND METHODS

### Chemicals and reagents

Polybrene and puromycin were purchased from Sigma-Aldrich (St. Louis, MO.). Antibodies for phosphorylated (“p”)-Akt (Ser-473) (9271), Akt1 (2967), p-p44/42 MAPK (Erk1/2) (9101) and Erk1/2 (9102), β-tubulin (2146), cyclin D1 (2922), cdc2 (77055), cleaved Caspase-3 (9661), cleaved Caspase-9 (20750), cleaved-PARP (5625), as well as EGFR (2232), PDGFRβ (3175) and FGFR (3472) were provided by Cell Signaling Tech (Shanghai, China). The antibodies of Ninj2 (ab172627) and Ninj2 (ab85891) were from Abcam (Shanghai, China). Fetal bovine serum (FBS), Dulbecco’s modified Eagle’s medium (DMEM), antibiotics, and all other cell culture reagents were obtained from Gibco-BRL (Suzhou, China). TRIzol reagent and other agents for RNA assays were purchased from Thermo-Fisher (Shanghai, China). mRNA primers were designed and provided by OriGene (Beijing, China), listed in Table 1.

**Table 1:** Primers utilized in the study.

**qPCR primers**	
*GAPDH* Forward	5′-GTCGTGTGAACGGATTTG-3′
*GAPDH* Reverse	5′-AAGATGGTGATGGGCTTCC-3′
*Ninj1* Forward	5′-TCATCTCCATCTCCCTTGTGCT-3′
*Ninj1* Reverse	5′-AGTCCAGCTTGGCGTGCTT-3′
*Ninj2* Forward	5′-CATCCTCTCACTACTACACCACC-3′
*Ninj2* Reverse	5′-CTGGTTGAGTCGCCACTGCTTT-3′

### Cell culture

Established human glioma cell lines, A172 and U251MG, were provided by the Cell Bank of Shanghai Institute of Biological Science (Shanghai, China). A172 cells and U251MG cells were cultured in DMEM with 10% FBS. Primary human glioma cells, derived from two independent informed-consent human patients, were from Dr. Cao at Soochow University [10, 11, 26]. The primary glioma cells were named as “P1/P2”. The primary human astrocytes were provided by Dr. Cao as well. Primary human glioma cells and astrocytes were cultured as described early [10, 11, 26]. All protocols of the present study, according to Declaration of Helsinki, were approved by the Ethics Board of Nanjing Medical University.

### Human tissues

Ten (10) human glioma tissues (“T”) together with paired surrounding normal brain tissues (“N”) were from Dr. Cao at Soochow University [10, 11]. Tissues were immediately stored in liquid nitrogen. For biomedical analyses, tissues were homogenized by using the tissue lysis buffer with proteasome inhibitors (Beyotime Biotechnology, Wuxi, China). Written informed-consent was obtained from each participant.

### Quantitative real-time reverse transcriptase polymerase chain reaction (qPCR) assay

At 1.5×10^5^ cells per well human glioma cells or astrocytes were seeded into six-well plates. Following the treatments, TRIzol reagents were added to cultured cells to obtained total RNA. By using an ABI7600 Prism system, qPCR was performed through a SYBR Green PCR kit. Melt curve analysis was always performed to calculate the product melting temperature. The 2^−Δ*C*t^ method was utilized for the quantification of targeted mRNA (*Ninj1* and *Ninj2*), with *GAPDH* *mRNA* tested as an internal control.

### Western blotting and co-immunoprecipitation (Co-IP)

Western blotting was performed through a well-established protocol [27]. The same set of lysates were run on separate gels (sister gels) to test different proteins with different molecular weights. Tubulin was always tested as the loading control. The NIH ImageJ software was utilized to quantify the intensity of each protein band. For each condition, 1000 μg total cellular lysates of glioma cells were pre-cleared with IgA/G (“Beads”, 30 μL of each treatment, Sigma). The endogenous Ninj2 proteins in TCL was captured by an anti-Ninj2 antibody (Santa Cruz Biotech) overnight, followed by incubation with IgA/G “Beads” for another 6-8h. Ninj2-immunoprecipitated proteins were further tested by Western blotting assays.

### Ninj2 shRNA

Two Ninj2 shRNAs, targeting non-overlapping sequences of Ninj2 (Seq1: GGAGCCTGGAGGAGCC CACGCAG and Seq2: CCCATCAACCTGAACCATT ACGC), were individually sub-cloned into the pLKO1-puro-GFP vector (Genepharm, Shanghai, China). The shRNA construct along with the lentivirus packaging plasmids (from Shanghai Genechem Co.) were co-transfected to HEK-293 cells, generating shRNA lentivirus. The virus was filtered, enriched and added to glioma cells (cultured in complete medium with polybrene). Afterwards, cells were cultured in the fresh complete medium for another 24h. Puromycin (2.5 μg/mL) was added for 4-5 passages, selecting stable cell lines. Control cells were infected with lentivirus with scramble control shRNA (Shanghai Genechem Co.). Ninj2 knockdown in the stable cells was always verified by Western blotting and qPCR.

### Ninj2 knockout

At 2×10^5^ cells per well A172 glioma cells were seeded into six-well plates. A lenti-CRISPR/Cas9-GFP-puro-Ninj2 KO construct (with targeted DNA sequence, 5′-GCATGGCGTTGGACATGAAC-3′) was provided by Dr. Zhang [19], added to A172 cells. GFP-positive A172 cells were first sorted by FACS, and single cells were cultured for another two weeks. The stable cells were further selected by puromycin-containing medium for 2-3 passages. In the stable A172 cells Ninj2 knockout was verified by Western blotting and qPCR.

### Ninj2 overexpression

The Ninj2-expressing lentivirus (“LV-Ninj2”) was provided from Dr Zhang as well [19]. The lentivirus (MOI=20) was added to cultured glioma cells, followed by puromycin (2.5 μg/mL) selection of stable cells (4-5 passages). Ninj2 overexpression in the stable cells was verified by Western blotting and qPCR.

### Cell apoptosis analyses

Human glioma cells with applied genetic modifications were seeded into six-well plates at 2×10^5^ cells per well. After culture for the applied time periods, cell apoptosis was examined by Annexin V-PI FACS, caspase-3 activity, TUNEL [terminal dexynucleotidyl transferase(TdT)-mediated dUTP nick end labeling] staining assays. TUNEL ratio (vs. DAPI) was calculated, recording 500 cells of each treatment from five random views (1:100 magnification). The protocols were described in detail in other studies [28].

### Cell proliferation and cell cycle assays

The detailed protocols of proliferation assays, including soft agar colony formation, BrdU ELISA, and EdU staining, as well as the propidium iodide (PI) FACS cell cycle distribution assays were reported early [29, 30].

### *In vitro* invasion and migration assays

As described [28], the “Transwell” chambers (Corning Co., New York, NY) with 12 μm pore were pre-coated with or without 1 mg/mL Matrigel (BD Biosciences, Shanghai, China). Human glioma cells were starved overnight, and added to the upper chamber (4 × 10^4^ cells of each chamber) in serum-free medium. The lower chamber was filled with completed medium with 10% FBS. After 24h incubation, cells that invaded/migrated to the lower chamber were fixed, stained and counted. Mitomycin (2.5 μg/mL Sigma) was included to exclude the influence of cell proliferation.

### Statistical analysis

For all *in vitro* experiment, n=5. Data of all repeated experiments were pulled together to calculate mean ± standard deviation (SD). Data were analyzed by one-way ANOVA followed by a Scheffe’s f-test by using the SPSS 18.0 software (SPSS Inc., Chicago, IL). A two-tailed unpaired T test (Excel 2017) was utilized to examine significance between two treatment groups. Significance was chosen as *P* < 0.05.
